# Role of small intronic RNAs in the crosstalk between immune cells and β-cells during type 1 diabetes development

**DOI:** 10.1080/15476286.2026.2645442

**Published:** 2026-03-13

**Authors:** Shagun Poddar, Flora Brozzi, Cristina Cosentino, Cécile Jacovetti, Claudiane Guay, Jérôme Perrard, Romano Regazzi

**Affiliations:** a Department of Fundamental Neurosciences, University of Lausanne, Lausanne, Switzerland; b Department of Biomedical Sciences, University of Lausanne, Lausanne, Switzerland

**Keywords:** Diabetes, islet, non-coding RNA, apoptosis, extracellular vesicles

## Abstract

Small non-coding RNAs, such as microRNAs and tRNA-derived fragments, are key regulators of cellular processes, but the functions of small intronic RNAs (sinRNAs), a recently identified RNA class, remain largely unknown. Here, we report that two sinRNAs, sinR-D and sinR-T, are upregulated in pancreatic β-cells of NOD mice, a well-established model of type 1 diabetes. Using in vivo RNA-tagging, we demonstrate that these sinRNAs are packaged into extracellular vesicles released by infiltrating CD4^+^ T lymphocytes and subsequently delivered to β-cells during the early stages of autoimmune attack. Functional analyses revealed that overexpression of sinR-T has little effect on β-cell viability, whereas sinR-D markedly increases β-cell apoptosis. This finding suggests that the transfer of sinR-D contributes to β-cell destruction and the onset of type 1 diabetes. Furthermore, pull-down experiments with biotinylated sinRNAs identified Ago2, a core component of the RNA-induced silencing complex (RISC), as a binding partner of sinR-D, indicating mechanistic parallels with microRNA-mediated regulation. Collectively, our data uncover a novel role for sinRNAs as extracellularly transferred regulators of β-cell fate, expanding the repertoire of small RNAs implicated in the initiation of type 1 diabetes.

## 1. Introduction

Type 1 diabetes (T1D) is an autoimmune disorder characterized by progressive loss of pancreatic β-cells, resulting in insulin deficiency and chronically elevated blood glucose levels [1,2]. The autoimmune attack is initiated by the infiltration of the islets of Langerhans by immune cells that release inflammatory mediators causing dysfunction and apoptosis of β-cells [3]. A better understanding of these events is necessary to permit the development of novel strategies to prevent and treat T1D.

Different classes of non-coding RNAs (ncRNAs), including microRNA (miRNAs), tRNA-derived fragments (tRFs), long non-coding RNAs and circular RNAs, contribute to the control of β-cell function and have been proposed to be involved in the development of various forms of diabetes [4–8]. Moreover, several circulating miRNAs have been proposed as biomarkers for T1D and its complications [9–12]. The level of several ncRNAs is altered in the islets of prediabetic NOD mice, a well-characterized model of T1D [13–15]. Many of these changes are triggered by chronic exposure of β-cells to proinflammatory cytokines, such as IL-1β, TNFα and IFNγ [16–19]. In addition, we found that a group of miRNAs produced by CD4^+^ T lymphocytes invading the islets can be directly transferred to β-cells via extracellular vesicles (EVs) [20]. Blockade of these miRNAs in the receiving β-cells permitted to partially prevent T1D occurrence in NOD mice, suggesting that this mechanism contributes to the development of the disease. Using an RNA-tagging approach, we recently reported that beside miRNAs, different tRFs are also shuttled from immune cells to β-cells, promoting apoptosis of insulin-secreting cells [21].

In addition to tRFs, this approach led to the identification of other small RNAs shuttling from T lymphocytes to β-cells. These include a group of small intron-derived RNAs (sinRNAs), a newly discovered class of non-coding RNAs distinct from mirtrons [22] originating from discrete genomic loci and reproducibly detected across independent high-throughput sequencing [23]. The purpose of the present study was to determine the functional role of these small ncRNAs in β-cells and to elucidate their mode of action. Our data further expand the repertoire of small non-coding RNAs regulating the activity of insulin-secreting cells and suggest a possible contribution of sinRNAs in T1D diabetes pathogenesis.

## 2. Materials and methods

### 2.1. Animals

ARRIVE guidelines were followed. Male C57BL/6NRj mice (aged 12–14 weeks) and female NOD.Cg-Prkdc scid/Rj (aged 4 weeks and 8 weeks) were purchased from Janvier while 8 weeks old female NOD BDC2.5 mice (NOD.Cg-Tg (TcraBDC2.5, TcrbBDC2.5)1Doi/DoiJ) were from Jackson Laboratory. The mice were housed on a 12-h light/dark cycle under standard conditions with *ad-libitum* chow diet and water access. All procedures were performed in agreement with the NIH guidelines and according to the Swiss national legislation and they were approved by the Swiss federal food safety and veterinary offices (animal licence numbers VD2744x3 and VD2495x4). Animal euthanasia has been performed by pentobarbital injection in accordance with the Swiss veterinary guidelines.

### 2.2. Cell lines

The murine MIN6B1 cell line was maintained in DMEM-GlutaMAX containing 25 mM glucose and 4 mM L-glutamine medium supplemented with 15% FBS, 70 µM β-mercaptoethanol, 100 U/mL penicillin and 100 μg/mL streptomycin. MIN6B1 cells were cultured in humidified 5% (vol/vol) CO_2_, 95% (vol/vol) air at 37°C and tested negative for mycoplasma contamination.

### 2.3. T lymphocyte isolation and cell culture

Mouse CD4^+^/CD25^−^ T-cells were purified from spleen of C57BL/6NRj mice using CD4^+^/CD25^−^ Regulatory T Cell Isolation Kit (Miltenyi Biotec, Germany). CD4^+^/CD25^–^ T cells were cultured in RPMI 1640 medium containing 10% EV-depleted FBS, 100 µg/mL streptomycin and 100 IU/mL penicillin, and stimulated with 20 ng/mL IL-12, 200 IU/mL IL-2, 2 μg/ml anti-CD28 and 5 μg/ml anti-CD3. Media and cells were harvested after 3 and 7 days for EV isolation and/or RNA extraction.

### 2.4. Isolation of pancreatic islets and β-cell sorting

Mouse islets were isolated as previously described [24] by digesting the pancreas with collagenase, followed by separation using Histopaque density gradient and handpicking. Islet cells were dispersed by incubation with Ca^2+^/Mg^2+^ free phosphate buffered saline, 3 mM EGTA and 0.025% trypsin for 2–3 min at 37°C and were then cultured in RPMI 1640 GlutaMAX medium supplemented with 10% FBS, 1 mM sodium pyruvate, 100 U/mL penicillin and 100 μg/mL streptomycin, and 10 mM Hepes, as previously described [20,21]. Sample preparations from NOD/ShiLtJ (aged 4 and 8 weeks, Janvier) and FAC (Fluorescence- Activated Cell) – sorted β-cells have been previously described [21]. Briefly, immune cells were first depleted with an anti-CD45 antibody and then β-cells were enriched from CD45-negative cells by autofluorescence. To achieve this goal, dissociated islet cells were first washed with FACS buffer (0.1% BSA, 2 mM EDTA, 11 mM glucose in PBS) and exposed for 5 minutes to TruStain FcX™ (anti-mouse CD16/32) Antibody (BioLegend), at 4°C. Then, cells were incubated for 30 min in the dark at 4°C with the following antibodies: 1:200 of FITC anti-CD45 (BioLegend 157,213), 1:40 of APC anti-CD3 (BioLegend 100,235), 1:20 of Brilliant Violet 421 anti-CD4 (BioLegend 100,437), and 1:20 of PE anti-CD25 (BioLegend 102,007). Cells were then washed twice with FACS buffer and analysed by FCF-Aria-II (SORP). On average, β-cell-enriched fractions contained 99.1 ± 0.9% insulin-positive cells and 0.6 ± 0.6% glucagon-positive cells.

### 2.5. Isolation of extracellular vesicles

EVs were isolated using a method described previously [20,21]. Briefly, the culture media (containing EV-depleted FBS) from mouse T cells were first centrifuged at 300 ×g for 6 minutes, and then at 2000 ×g for 10 minutes. The supernatants were then centrifuged at 10,000 ×g for 30 minutes to eliminate cell debris, and subsequently at 100,000 ×g for 2 hours. The resulting pellets containing the EVs were washed twice with PBS and subjected to a final centrifugation at 100,000 ×g for 2 hours.

### 2.6. Cell transfection and treatment

Dispersed mouse islet cells were transfected with scrambled or custom-designed RNA oligonucleotides (IDT) mimicking the sequence of sinR-D2/D3 or sinR-T using Lipofectamine 2000 and incubated for 48 h before RNA extraction or functional assays. Oligonucleotide sequences are provided in Supplementary Table S2. The effect of pro-inflammatory cytokines was examined by incubating the cells with IL-1β (0.1 ng/mL), TNF-α (10 ng/mL), and IFN-γ (30 ng/mL) for 24 h. For the treatment with EVs, islet cells were exposed to EVs (1x10^11^/mL) for 24 h.

### 2.7. Argonaute 2 immunoprecipitation

MIN6B1 cells were transfected using with a plasmid encoding Flag-tagged Ago2. They were then detached using trypsin, washed, pelleted, and stored at −80°C until needed. The cell pellets were homogenized in 500 µL of lysis buffer (1% Triton X-100, 150 mM NaCl, 10 mM Tris pH 7.5, 1 mM EDTA), supplemented with a protease inhibitor cocktail, 0.16 U/µL RNase inhibitor, and 0.5 mM DTT. After 15 minutes incubation, the lysates were centrifuged at 16,000xg for 10 minutes at 4°C. Protein concentrations were determined by Bradford assay. Equal amounts of protein extracts were incubated with 20 µL equilibrated anti-Flag magnetic beads (Sigma M8823) for 2 hours at 4°C under agitation. Beads were isolated using a magnet and washed eight times (five washes with lysis buffer and three washes with PBS). One fifth of the beads was incubated in 1X Laemmli buffer to measure protein content while the rest was used for RNA extraction.

### 2.8. Immunofluorescence

The cells were cultured in removable 12-well chamber slides (Cat # 81,201, Ibidi, Germany) coated with Poly-L-Lysine and laminin, and were fixed with methanol. They were then permeabilized using saponin and double-stained with 1:200 mouse anti-insulin (cat # 66,198–1, Proteintech, USA) and 1:500 rabbit anti-cleaved-caspase 3 (cat #9661S, Cell Signaling Technology, USA) antibodies. AlexaFluor 488 anti-mouse and AlexaFluor 555 anti-rabbit (1:400, Invitrogen, USA) were used as secondary antibodies. Cell nuclei were stained with Hoechst 33,342 (1 μg/ml, Invitrogen). Coverslips were mounted on microscope glass slides with Fluor-Save mounting medium (VWR International SA). The images were acquired with a Zeiss Axiovision fluorescence microscope.

### 2.9. Insulin secretion and insulin ELISA

Transfected mice islet cells and MIN6 cells were first incubated at 37°C for 60 min in a Krebs – Ringer bicarbonate buffer (KRBH) containing 25 mM HEPES, pH 7.4, 0.1% BSA, and 2 mM glucose. Thereafter, the cells were incubated at 37°C for 45 min in KRBH-BSA solutions with 2 mM (basal) or 20 mM (stimulatory) glucose. After incubation, supernatants were collected. The cells kept at basal glucose were harvested using acid ethanol (75% ethanol, 0.55% HCl), and those incubated at stimulatory conditions were lysed using Triton X-100 lysis buffer to determine insulin and protein contents, respectively. Insulin levels were measured by ELISA according to the manufacturer’s protocol (Mercodia, Sweden) and cellular protein contents by Bradford assay.

### 2.10. Pull-down assay and western blotting

To elucidate the interaction between sinRNAs and Ago2, a pull-down assay was performed using 5’- biotinylated oligonucleotides mimicking the sequence of sinR-D or sinR-T or their scrambled controls as previously described [25]. Briefly, 1 mg of MIN6B1 cell lysate was prepared using lysis buffer (50 mM Tris – HCl pH 7.5, 150 mM NaCl, 1% NP40, 0.1% SDS, 0.5% sodium deoxycholate, 1 mM DTT, protease inhibitor cocktail, 80 U/ml RNase inhibitor) and incubated with 3 µg of oligonucleotides for 1 h at room temperature on a vertical rotator. Further, 50 μL of streptavidin dynabeads (*M*-280 Streptavidin, 11206D, Invitrogen) was added to the samples and incubated again for 1 h at room temperature on a vertical rotator. The beads were thoroughly washed with a solution containing 50 mM Tris-HCl (pH 7.5), 150 mM NaCl, 1% NP-40, 0.1% SDS, and 0.5% sodium deoxycholate. Subsequently, the proteins were eluted by heating the samples in Laemmli buffer at 95°C for 5 minutes. Protein extracts were loaded on SDS-PAGE and transferred to nitrocellulose membranes (BioRad). The membranes were blocked using 5% BSA in TBST buffer and then incubated with rabbit anti-Ago2 antibody (cat# ab186733, Abcam, UK), overnight at 4°C followed by incubation for 1 h at RT with the secondary antibody AlexaFluor 555 anti-rabbit (1:400, Invitrogen, USA). The blots were developed using ECL substrate (cat# 34,075, Thermo Fisher, USA).

### 2.11. Small RNA-seq bioinformatics analysis

Sequencing data was filtered to retain reads between 16 and 55 nucleotides in length as previously described [21]. Sequences were further filtered to include only those with a minimum of 5 counts in at least 4 samples, yielding 63,367 unique sequences. To focus on mouse-derived fragments and exclude contaminants or sequences of unknown origin, only reads matching the reference *Mus musculus* genome (mm10) were retained. Differential expression analysis was conducted using DESeq2 on three sets of sequences: all 7900 fragments, 242 tRNA-derived fragments, and 217 microRNA-derived fragments [21] with normalization validated through miR-238 *C. elegans* spike-in control. Statistical significance was determined using an adjusted *p*-value threshold of ≤0.01, identifying differentially abundant fragments in EU-tagged RNA extracted from receiving β-cells.

### 2.12. Proteomics analyses

All raw MS data together with raw output tables are available via the Proteomexchange data repository (www.proteomexchange.org) with the accession P×D065257. Samples were digested following the SP3 method [26] using magnetic Sera-Mag Speedbeads (Cytiva 45,152,105,050,250, 50 mg/ml). Briefly, samples were diluted with SP3 buffer (2% SDS, 10 mM DTT, 50 mM Tris, pH 7.5) and heated 10 min at 75°C. Proteins were then alkylated with 30 mM iodoacetamide for 45 min at RT in the dark. Proteins were precipitated on beads with ethanol (final concentration: 60%). After 3 washes with 80% ethanol, beads were digested in 50 ul of 100 mM ammonium bicarbonate with 1.0 ug of trypsin (Promega #V5113). After 1 h of incubation at 37°C, the same amount of trypsin was added to the samples for an additional 1 h of incubation. Supernatants were then recovered and two sample volumes of isopropanol containing 1% TFA were added to the digests. The samples were then desalted on a strong cation exchange (SCX) plate (Oasis MCX; Waters Corp., Milford, MA) by centrifugation to remove traces of SDS. After washing with isopropanol/1%TFA and 2% acetonitrile/0.1% FA, peptides were eluted in 200 ul of 40% MeCN, 59% water, 1% (v/v) ammonia, and dried by centrifugal evaporation. LC-MS/MS analyses were carried out on a TIMS-TOF Pro (Bruker, Bremen, Germany) mass spectrometer interfaced through a nanospray ion source to an EvoSep One liquid chromatography system (EvoSep, Odense, Denmark). Peptides were separated on a reversed-phase Aurora Elite C18 column (15 cm, 75 μm ID, 1.7um, IonOpticks) at a flow rate of 200 nl/min. Data-independent acquisition was carried out using a method similar to the DIA-PASEF method reported previously [27]. Identification of peptides directly from DIA data was performed with Spectronaut 19.9 with the Pulsar engine using the ‘deep’ setting and searching the reference mouse proteome (www.uniprot.org) database of 6 February 2025 (54’739 sequences), and a contaminant database containing the most usual environmental contaminants and enzymes used for digestion [28]. For identification, peptides of 7–52 AA length were considered, cleaved with trypsin/P specificity and a maximum of 2 missed cleavages. Carbamidomethylation of cysteine, methionine oxidation and N-terminal protein acetylation were the modifications applied. Ion mobility for peptides was predicted using a deep neural network and used in scoring.

Peptide-centric analysis of DIA data was done with Spectronaut 19.9 using the library generated by Pulsar from DIA data. Peptide quantitation was based on XIC area, for which a minimum of 1 and a maximum of 3 precursors were considered for each peptide, from which the mean value was selected. Quantities for protein groups were derived from inter-run peptide ratios based on MaxLFQ algorithm [29]. Global normalization of runs/samples was done based on the median of peptides. All subsequent analyses were done with an in house developed software tool (https://github.com/UNIL-PAF/taram-backend). Contaminant proteins were removed, and quantity values for protein groups generated by Spectronaut were log2-transformed. Missing values were imputed based on a normal distribution with a width of 0.3 standard deviations (SD), down-shifted by 1.8 SD relative to the median.

### 2.13. Statistical analysis

Data are presented as means ± SD. A two-tailed one-sample t-test was conducted to compare the datasets against a control value set at 1. Unpaired t-tests were used for comparing two datasets. One-way Anova followed by Dunnett post hoc test was used for multiple dataset comparison. Differences between datasets were considered statistically significant if the *p*-value was less than 0.05.

## 3. Results

**Figure 1.**
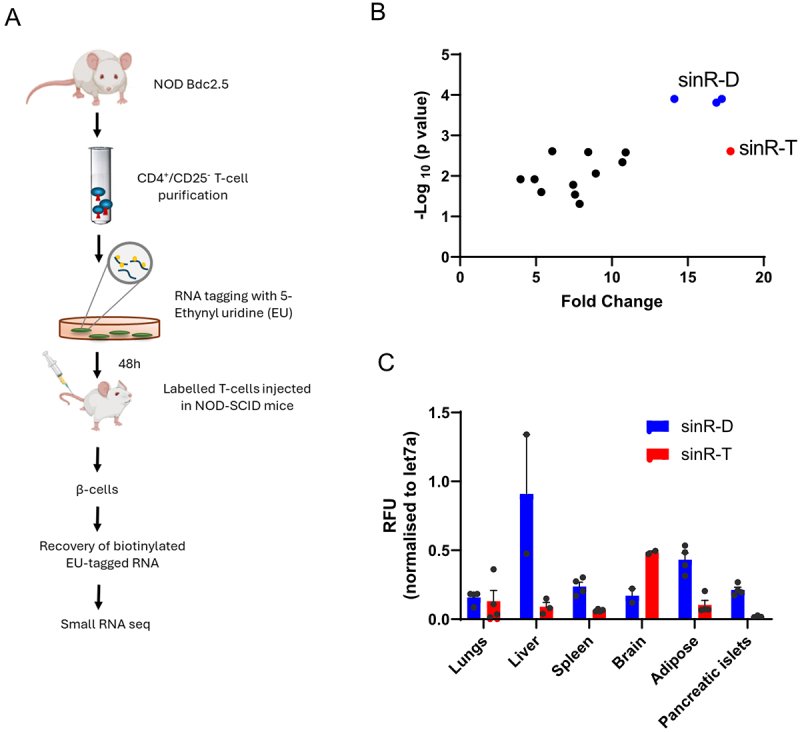
Identification of small RNAs transferred *in vivo* from CD4^+^/CD25^−^ T cells to pancreatic β-cells. (A) schematic representation of *in vivo* T cell adoptive transfer and identification of EU-tagged RNA in pancreatic β-cells. As previously described [21], CD4^+^/CD25^−^ T cells were purified from NOD.Cg-tg (TcraBDC2.5, TcrbBDC2.5) mice and were incubated with 200 μM ethynyl uridine (EU) for 48 h. The labelled T cells were injected in the tail vein of NOD.CB-17-Prkdc.Scid/Rj mice. Saline solution was injected intravenously as control. The pancreatic islets were isolated after 48 h and β-cells purified by FACS. EU-tagged RNAs were biotinylated using a Click-iT nascent RNA capture kit and then isolated on streptavidin-coated beads. The eluted RNA was used for library preparation and small RNA sequencing. (B) plot showing differential expression of small RNA fragments (16–29 nucleotides) in EU-tagged RNA extracted from the receiving β-cells. Each point represents a small RNA fragment, with the x-axis displaying Fold changes and the y-axis -log_10_ adjusted *p*-values. Blue points highlight the three upregulated sequences of sinR-D and the redpoint sinR-T. As internal control, *C. elegans* miR-238 RNA sequence was spiked into each sample, and its EU-tagged sequence was used for normalization across samples. (C) to demonstrate widespread expression of the selected sinRNAs, tissue samples were collected from C57BL6 mice and sinRNA levels were measured using qRT-PCR. The relative abundance of sinRnas in each tissue was normalised to Let7a. The results are expressed as relative fluorescent units (RFU).

NOD mice are a well-known T1D model characterized by progressive infiltration of immune cells in the islets of Langerhans, causing β-cell loss and culminating in diabetes appearance [13–15,30]. We recently reported that during the initial phases of T1D, different tRFs are transferred from immune cells to insulin-secreting cells, potentially contributing to β-cell elimination and to the development of the disease [21]. To identify the RNAs transferred to β-cells during the autoimmune attack, CD4^+^ T-cells isolated from NOD BDC2.5 mice were incubated with 5’-ethynyl uridine (EU), a nucleotide derivative incorporated in RNAs during transcription. Upon inoculation into NOD.SCID mice, CD4^+^ T-cells from NOD BDC2.5 mice trigger the appearance of autoimmune diabetes [31]. Two days after the adoptive transfer, when the receiving mice were still in the initial phases of the disease, β-cells were isolated by FACS and EU-tagged RNA molecules shuttled from CD4^+^ T cell to β-cells were purified on streptavidin beads. A schematic representation of the experiment can be found in Figure 1(A). In addition to the previously studied tRFs [21], bioinformatic analysis of the RNAs shuttling from T lymphocytes to β-cells revealed an enrichment of small intron-derived RNAs (sinRNAs) (Supplementary table S1; Figure 1(B)). The three RNAs most significantly transferred to β-cells listed as sinR-D1, sinR-D2 and sinR-D3 map to an intronic region of the *Dnaaf1* gene (Figure S1A). This sequence is part of an active enhancer validated by STARR-seq (Self-Transcribing Active Regulatory Region sequencing) [32]. Sequence alignment revealed that sinR-D1, D2, and D3 share a conserved 16-nt core sequence (CTCGGTAGAACCTCCA), with differences restricted to one or two nucleotides at the 5′ end. Given this minimal variability and their origin from the same intronic region of the *Dnaaf1* gene, we inferred a shared biogenesis. Therefore, for downstream detection and quantification assays, we selected the sinR-D3-specific primer, as it provides broad coverage of this sinRNA family while avoiding redundancy in primer design. Another interesting candidate is sinR-T which is 24 nt in length and maps to the intronic region of the *Ttll11* gene (Figure S1B). These sinRNAs are detectable in various tissues (Figure 1(C)), suggesting that their expression is not restricted to immune cells and β-cells. To verify the results obtained by small RNA-seq, we performed qRT-PCR analysis on the EU-tagged RNAs released by CD4^+^ T lymphocytes and recovered in β-cells two days after the adoptive transfer of the immune cells. The results obtained confirmed the finding of the RNA-seq (Figure S2).

**Figure 2.**
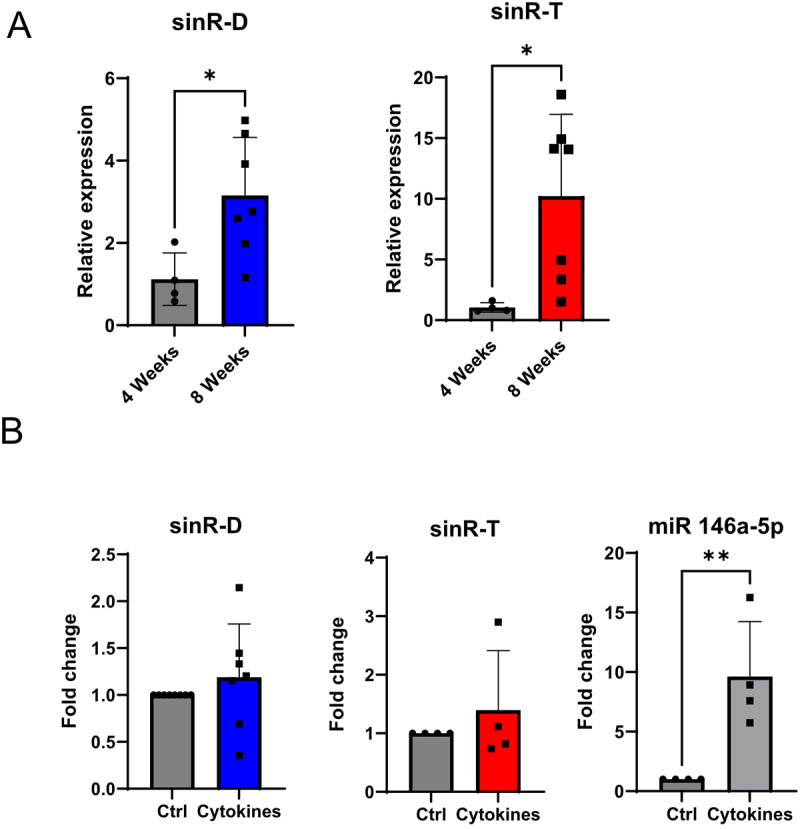
Levels of sinRNAs in prediabetic NOD mice and cytokine-treated islets. (A) sinRNA expression levels were quantified in NOD mice at 4 weeks and 8 weeks by qRT-PCR, with miR-184 serving as the normalization control. Data shown are the means ± SD, *n* = 4–7, *p < 0.05. (B) Islet cells of C57BL/6NRj mice incubated with or without cytokines (IL-1β, IFN-γ and TNF-α) for 24 h prior to RNA extraction. qRT-PCR was performed to measure sinRNA levels, with Let7a used for normalization (*n* = 4–7 independent experiments) mean ± SD.

As these sinRNAs are transferred from immune cells to β-cells, we examined whether their level is increased in the islets of prediabetic NOD mice (8 weeks old) compared to the islets of young NOD mice (4 weeks old) that are devoid of insulitis [33]. As shown in Figure 2(A), the levels of sinR-D3 and sinR-T were increased significantly in the islets of prediabetic NOD mice where immune cells have begun to invade the islets. To rule out age-related effects, we also assessed sinRNA levels in age-matched NOD-SCID mice at 4 and 8 weeks of age, which do not develop diabetes due to the absence of functional immune cells. Notably, no significant changes in sinR-D3 or sinR-T levels were observed in NOD-SCID islets (Figure S3), suggesting that the upregulation is associated with immune infiltration rather than ageing.

Proinflammatory cytokines released by the infiltrating immune cells play a crucial role in regulating the expression of different genes and small ncRNAs in β-cells [20,21]. To examine this possibility, we checked the effect of proinflammatory cytokines on the levels of these sinRNAs. Pancreatic islets isolated from C57BL6N mice were dissociated and treated with a mix of proinflammatory cytokines (IL-1β, TNF-α and IFN-γ). After 24 h incubation, RNA was extracted and the level of the sinRNAs measured by qRT-PCR. We found that, in contrast to the positive control miR-146a, the level of these sinRNAs was not significantly affected by cytokine exposure (Figure 2(B)), suggesting that the increase observed in β-cells after the adoptive transfer of CD4^+^ T cells and in the islets of prediabetic NOD mice is not caused by the presence of proinflammatory cytokines.

**Figure 3.**
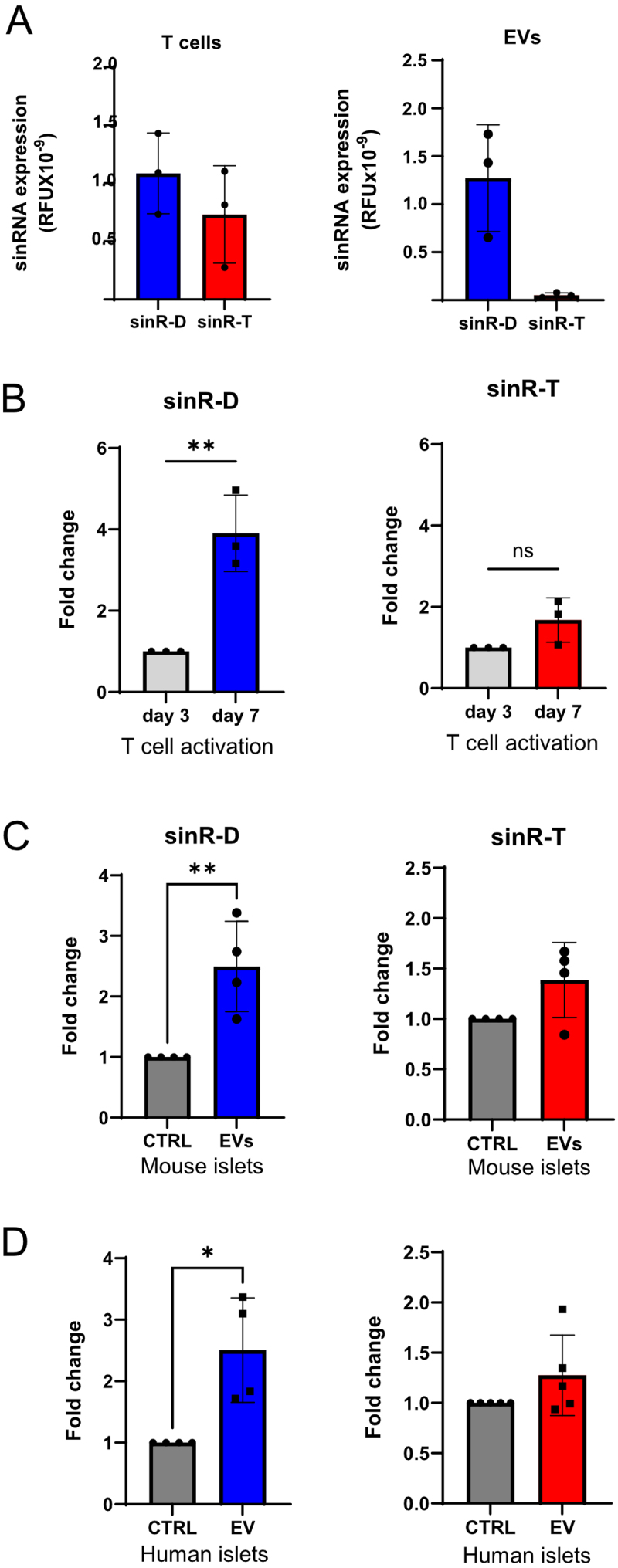
Transfer of sinRNAs from T cells to islet cells via extracellular vesicles. (A) Expression levels of sinRNAs were measured in 100ng RNA extracted from activated CD4^+^/CD25^−^ T lymphocytes and from EVs released by these cells. The values are calculated using 2^^-Ct^ method and presented as relative fluorescent units (RFU). (B) RNA was extracted from CD4^+^/CD25^−^ T lymphocytes following activation of 3 days (3d) and 7 days (7d) and the levels of the sinRNAs measured by qPCR using Let7a for normalisation (*n* = 3). (C-D) qRT-PCR analysis of sinRNA levels in pancreatic islet cells following a 24-hour incubation with extracellular vesicles (EVs) released by CD4^+^ T cells. (C) Mouse islets incubated with EVs from murine CD4^+^ T cells and (D) Human islets with EVs from CD4^+^ T cells of human donors. Let7a was used for normalization. Data are presented as mean ± SD, with *n* = 4 biological replicates. Statistical significance was determined as **p < 0.01.

EVs mediate the exchange of signals between different cell types [34]. T cells secrete EVs with distinct cargoes, including miRNAs that contribute to increase the apoptotic rate of recipient islet cells [20]. The expression of sinR-D3 and -T was detected both in the RNA extracted from activated CD4^+^/CD25^−^ T lymphocytes and in the EVs released by them (Figure 3(A)). Moreover, the levels of sinRNAs were increased in a time-dependent manner upon activation of CD4^+^/CD25^−^ T lymphocytes as shown in Figure 3(B). To investigate whether the EVs released by T cells could mediate the rise of sinRNAs in islet cells, CD4^+^/CD25^−^ T lymphocytes were extracted from the spleen of C57BL6N mice and were activated with IL2, IL12 and anti-CD3 and anti-CD28 beads. The EVs released from T lymphocytes were then purified by ultra-centrifugation and added to the culture medium of dispersed mouse islet cells. After 24 h, RNA was extracted and the level of the sinRNAs was assessed by qRT-PCR. These measurements showed that the level of the sinR-D3 in islet cells is increased in the presence of the EVs released by activated CD4^+^/CD25^−^ T lymphocytes (Figure 3(C)). Although a similar trend was observed for sinR-T, the effect was smaller and did not reach statistical significance. To evaluate whether this process also occurs in humans, human islet cells were incubated with exosomes (EV-hT) derived from CD4^+^ T cells isolated from human blood donors, as previously described [20]. Consistent with the findings in mice, exposure of human islet cells to EV-hT facilitated the transfer of sinR-D3, whereas transfer of sinR-T was comparatively limited (Figure 3(D)). Taken together, these in vitro findings support our previous *in vivo* results demonstrating that sinRNAs are transferred from T lymphocytes to pancreatic islet cells via EVs during the development of T1D.

**Figure 4.**
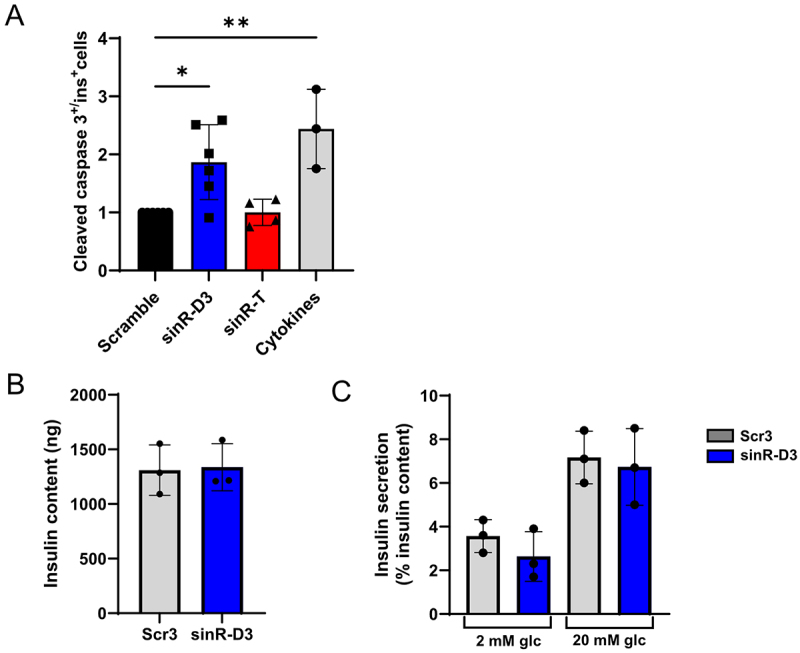
Functional impact of sinRNAs on pancreatic β-cells. (A) Mouse islet cells were transfected with sinRNA mimics or with a scrambled oligonucleotide sequence and incubated for 48 h. Where indicated, islet cells were exposed to pro-inflammatory cytokines (IL-1β, IFN-γ, and TNF-α) for 24 h. At the end of the incubation, the coverslips were stained with anti-insulin and anti-cleaved caspase-3 antibodies, and the percentage of cleaved caspase-3 positive β-cells was determined. Fold changes were then calculated relative to the scrambled condition. *p < 0.05, *n* = 3–6, mean ± SD. (B) Insulin content of mouse islets transfected with sinR-D3 or with a scrambled oligonucleotide sequence (Scr3). (C) Glucose-induced insulin release of transfected mouse islet cells (as mentioned above) in response to 2 mM glucose (basal) or to 20 mM glucose (stimulatory).

As demonstrated in our previous work [20,21], exposure to T cell-derived EVs leads to β-cell death. We hypothesized that these sinRNAs may contribute to this effect upon their transfer. To investigate the functional relevance and the impact of these sinRNAs, we overexpressed them in mouse islet cells using oligonucleotides mimicking the sequence of sinR-D3 and sinR-T and we then assessed the impact on β-cell death (Figure S4). Immunofluorescence analysis with anti-insulin and anti-cleaved caspase-3 antibodies revealed a significant increase in apoptosis upon sinR-D3 over-expression (Figure 4(A)). In contrast, no significant difference was observed when the level of sinR-T was increased. These findings suggest that the rise of sinR-D3 resulting from the delivery of T cell EV cargoes may contribute to β-cell death occurring during the initial phases of the disease. Insulin content (Figure 4(B)) and glucose-induced insulin secretion (Figure 4(C)) were not affected by overexpression of sinR-D3. Similar findings were obtained by overexpressing sinR-D3 alone or in combination with sinR-D2 in MIN6 cells (Figure S5).

**Figure 5.**
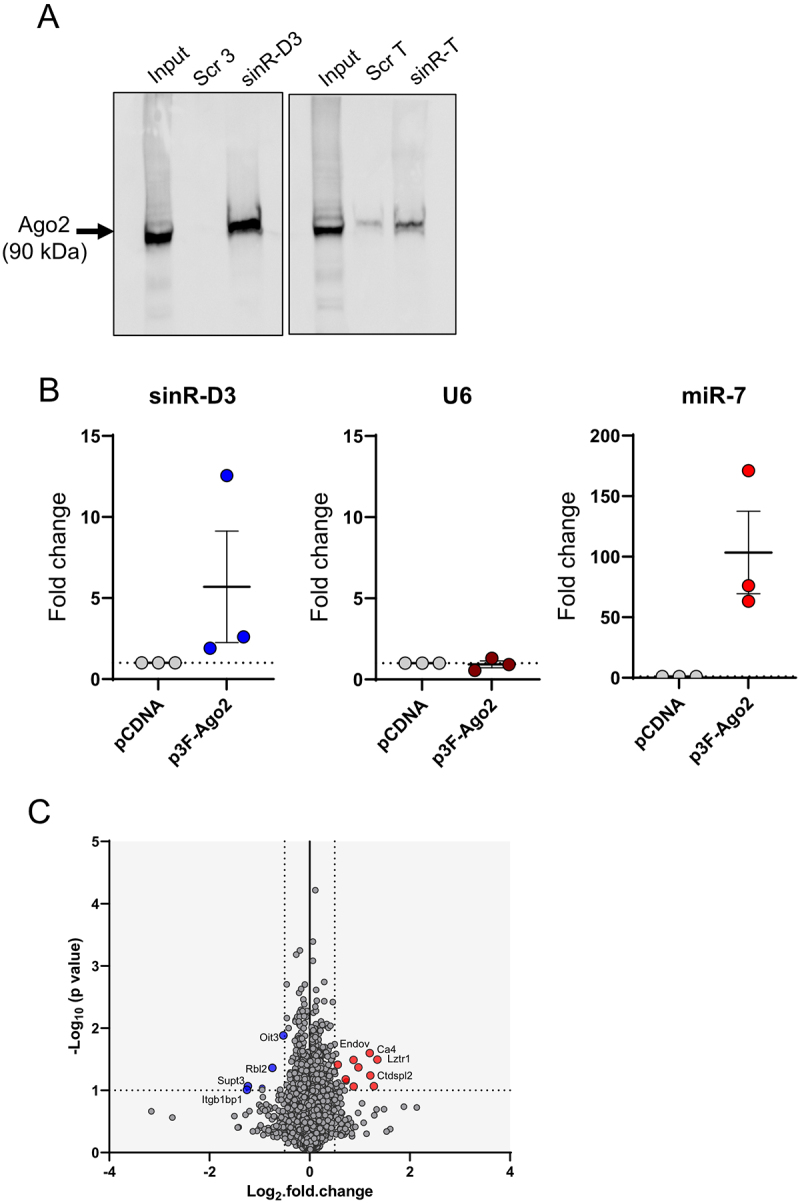
Mode of action of sinRNAs. (A) Western blot analysis demonstrating the enrichment of Ago2 protein in MIN6 cells incubated with either biotinylated sinRNA mimics or the corresponding scrambled control sequences, followed by pull-down with streptavidin beads. Input lane represents 10% of the total cell lysate. Data shown is a representative image from three independent experimental replicates. (B) MIN6 cells were transfected with a plasmid encoding Flag-tagged Ago2. The small non-coding RNAs immunoprecipitated using an anti-Flag antibody were analyzed by qRT-PCR. (C) volcano plot representing differentially expressed proteins identified by proteomic analysis of pancreatic islets overexpressing both sinR-D2 and sinR-D3 compared to control cells transfected with length-matched scrambled sequences. Significantly upregulated proteins are shown in red, and significantly downregulated proteins are shown in blue (*p* < 0.05).

Although the exact mechanism of action of sinRNAs remains largely unexplored, few reports suggest that some of them could bind Ago2 and act as miRNAs [35] and other small RNA classes [36–38]. Indeed, evidence for the physical association of some sinRNAs with Ago2 and for their capacity to regulate gene expression has been recently reported [23]. Notably, we found exact sequence matches for sinR-D2 and sinR-D3 in publicly available datasets from the same study [23], specifically in Supplementary Data 7 (Top 5000 15–30 nt Ago2-binding sRNA-OHs identified by TANT-seq in Hepa 1–6 cells) and Supplementary Data 9 (Top 5000 15–30 nt Ago2-binding sRNA-OHs identified by TANT-seq in human 293T cells). These findings support the incorporation of sinR-D2 and sinR-D3 into Argonaute complexes and suggest a conserved potential for functional activity across species and cell types. To verify whether sinR-D3 and sinR-T are able to bind to Ago2, we incubated biotinylated mimics of each sinRNA with lysates of the insulin-producing MIN6B1 cells (Figure S6). We then used streptavidin beads to isolate from the extract the proteins associated to these small RNAs. Western blot analysis using an antibody against Ago2 demonstrated a clear interaction between Ago2 and sinR-D3, while little or no specific interaction was observed with sinR-T (Figure 5(A)). To further confirm the interaction of sinR-D3 with Ago2, MIN6B1 cells were transfected with a plasmid encoding a FLAG-tagged Ago2 construct. Two days later, Ago2 was immunoprecipitated with an anti-FLAG antibody and the co-immunoprecipitated RNAs analysed by RT-PCR. As expected, both miR-7 and sinR-D3 were co-immunoprecipited with Ago2 but not U6, another small non-coding RNA that does not interact with Ago2 (Figure 5(B)).

Given the observed physical association of sinR-D3 with Ago2, we next aimed to assess the impact of this sinRNA on protein expression. To capture the full range of potential regulatory effects, we conducted overexpression experiments using a combined mixture of sinR-D2 and sinR-D3, which differ by a single nucleotide at the 5′ end. This choice was based on the principle that, similar to miRNAs, small RNA function can be highly sensitive to sequence variation, particularly within the seed region, where even a single nucleotide difference may alter target specificity [39–42]. By including both variants, we aimed to ensure comprehensive representation of potential target interactions. The resulting proteomic changes were then analysed by quantitative mass spectrometry. Proteomic analysis of sinRNA-overexpressing pancreatic islets revealed a downregulation of RBL2, OIT3, ITGB1BP1, and SUPT3, which are associated with pathways involved in cell cycle regulation, adhesion, and transcription [43–48]—processes whose disruption is known to promote apoptosis (Figure 5(C), Supplementary Table S3). Conversely, CA4, LZTR1, ENDOV, CTDSPL2, and ST3GAL5 were significantly upregulated and are linked to stress response, DNA damage, and apoptotic signalling [49–53], suggesting activation of pro-apoptotic mechanisms in response to sinRNA overexpression.

Collectively, these data suggest that, in addition to miRNAs and tRFs, distinct sinRNAs are also transferred from immune cells to pancreatic β-cells during the early phases of T1D. Among them, sinR-D3 was found to interact with Ago2 and may lead to gene regulation that contributes to β-cell death.

## 4. Discussion

The discovery of small ncRNAs has rapidly emerged as a transformative area in molecular biology. These small ncRNAs typically 15 to 50 nucleotides in length, include well-characterized species such as miRNAs. Recent advancements in high-throughput sequencing technologies and bioinformatics tools have expanded the repertoire of identified small ncRNAs across diverse species [23,54,55]. This progress has sparked growing interest in newer classes of small ncRNAs derived from longer, structured RNA molecules such as tRFs (derived from transfer RNAs) [56,57], rRFs (derived from ribosomal RNAs) [58], Y RNA fragments [59], small nuclear RNAs (snRNAs) [60], small nucleolar RNAs (snoRNAs) [61], vault RNAs (vtRNAs) [62], and other less-characterized small ncRNAs [23,63]. Understanding the functional roles of these emerging small ncRNAs is crucial to elucidating their involvement in physiological and pathological processes.

In this study, we investigated a subset of small ncRNAs derived from the cleavage of intronic sequences, focusing on their role in the early stages of T1D development. Many small ncRNAs are incorporated into EVs and are selectively released into the extracellular space, where they play an important role in intercellular communication [20,21,64,65]. Indeed, the delivery of small ncRNAs carried by EVs has been shown to trigger functional changes in the receiving cells and to contribute to many pathological processes, including the development of different forms of diabetes [66].

Recent evidence indicates that lymphocytes infiltrating the islets of Langerhans during T1D development release EVs containing a distinct set of miRNAs. These miRNAs are transferred to β-cells, triggering the activation of apoptotic pathways [20,67,68]. In addition to miRNAs, we recently demonstrated that EVs from CD4^+^ T cells invading the islets also transfer tRFs to β-cells [21]. This suggests that EVs molecular cargo extends beyond the known small ncRNAs, and RNA labelling methodologies are able to unveil unexplored molecules that contribute to cell-to-cell communication.

Our study focused on the functional role of two small RNAs, which are derived from intronic regions and are shuttled between CD4^+^ T cells and β-cells during the initial stages of T1D. Specifically, sinR-D3 originates from the host gene Dnaaf1, and sinR-T is derived from Ttll1. We found that these sinRNAs are expressed in various tissues, with elevated levels in pancreatic islets of prediabetic NOD mice, a well-established model of T1D. Notably, high-throughput sequencing studies have identified sequences corresponding to our sinRNAs in mouse and human cell lines [23], further validating their existence as an underappreciated class of small ncRNAs.

Functionally, we observed that overexpression of sinR-D3 induces apoptosis in pancreatic β-cells, suggesting a regulatory role in insulin-secreting cells and a potential contribution, together with other sncRNAs such as miRNAs and tRFs, to T1D progression through β-cell death. Although the level of sinR-D3 overexpression achieved is relatively high, the observed increase in β-cell apoptosis is unlikely to be a non-specific effect, as comparable overexpression of sinR-T does not significantly affect survival of insulin-secreting cells. Our transfection experiments represent a model system designed to evaluate whether sinRNAs may play a role in the regulation of β-cell function. However, this approach does not fully recapitulate in vivo conditions, where sinRNA levels will be elevated over several weeks rather than a few hours. While the precise molecular mechanisms triggering apoptosis remain to be fully elucidated, our evidence indicates that sinR-D3 may act similarly to miRNAs, mediating post-transcriptional gene repression. Indeed, pull-down experiments with biotinylated oligonucleotides revealed that sinR-D3 interacts with Ago2, a key component of the RISC complex. These findings align with the observations of Lai et al. [23], who demonstrated that many sinRNAs, including sinR-D, co-immunoprecipitate with Ago2. Although overexpression of sinR-D resulted in changes in the level of proteins potentially involved in apoptosis, the precise mechanisms through which this small ncRNA affects β-cell survival remains to be fully elucidated and would need to be addressed in the future. Some of the observed changes in protein expression may be directly mediated by sinR-D, while others may represent secondary effects of apoptosis induction. Therefore, although our data provide a global proteome landscape of sinR-D-overexpressing cells, they do not yet allow to establish a clear mechanistic link to the observed phenotype. A model describing the proposed contribution of sinR-D to β-cell apoptosis is shown in Figure S7.

Our study expands the repertoire of ncRNAs involved in the crosstalk between immune cells and β-cells, highlighting their regulatory roles in insulin-secreting cells under diabetic conditions. These findings underscore the need to investigate sinRNAs further to understand their contributions to cellular signalling pathways. A deeper understanding of this overlooked class of small ncRNAs could provide significant insights into the pathogenesis of diabetes and other human diseases.

## Supplementary Material

Supplemental material revision.pdf

## Data Availability

All the data are available upon request.
