# Supplementary material for: Role of small intronic RNAs in the crosstalk between immune cells and β-cells during type 1 diabetes development
Source: RNA Biol. 2026 Mar 13;23(1):1–15. doi: 10.1080/15476286.2026.2645442 (PMC12998025; doi:10.1080/15476286.2026.2645442)
Supplement: Supplemental material revision.pdf [file KRNB_A_2645442_SM1515.pdf]

hit	Fold Change	p-value	Adj. p-value	sequence	Length (nt)
sinR_D1	14,12	1,84E-08	1,25E-04	CTCGGTAGAACCTCCA	16
sinR_D2	17,24	3,20E-08	1,25E-04	ATCTCGGTAGAACCTCCA	18
sinR_D3	16,88	6,03E-08	1,56E-04	TCTCGGTAGAACCTCCA	17
sinR_T	17,82	1,69E-06	2,47E-03	GGGGATATAGCTCAGTGGTAGAGC	24
tRNA-Lys-CTT	6,07	1,91E-06	2,47E-03	GCCCGGCTAGCTCAGTCGGTAGAGCA	26
LncRNA (Gm38528)	8,44	2,33E-06	2,59E-03	CCTGTTCGCAGCGCCA	16
AC108813.8	10,91	2,73E-06	2,65E-03	CCACCTAGGGACGCCA	16
OK040659.1	10,70	5,24E-06	4,53E-03	CCCCGTCTCGGAGCCA	16
AL358892.13	8,94	1,36E-05	8,80E-03	ACTGAAGTGGAGAAGGG	17
AC112142.4	4,91	1,99E-05	1,19E-02	AGTCAGGTGGGGAGTT	16
piRNA2431	3,98	2,18E-05	1,21E-02	AATAGGGAACGTGAGCTGGGTTTAG	25
tRNA-Lys-CTT	7,45	3,20E-05	1,66E-02	GCCCGGCTAGCTCAGTCGGTAGAGC	25
LSU-rRNA	5,35	5,85E-05	2,53E-02	AGTCGGAATCCGCTAAGGAGTGTG	24
tRNA-Phe-GAA	7,57	6,99E-05	2,86E-02	GCCGAAATAGCTCAGTTGGGAGAG	24
SSU-rRNA	7,87	1,32E-04	4,89E-02	GGAGGGCAAGTCTGGTGCCAGCAGC CGCG	29

Supplementary Table 1) Most upregulated small ncRNA identified by RNA-seq analysis of pulled-down EU-tagged RNA in mice islets. $\log_2$ fold change > 2, adjusted *p-value* < 0.05

Oligo name	Sequence (5’-3’)
Scrambled for sinR-D2	GCUGACUACACCAUAGUC
sinR-D2 mimic	AUCUCGGUAGAACCUCCA
Scrambled for sinR-D3	GUCGCAACUCGUACAUC
sinR-D3 mimic	UCUCGGUAGAACCUCCA
sinR-T mimic	GGGGAUAUAGCUCAGUGGUAGAGC
Scrambled for sinR-D	bio-CGAAAAUUGUUCACCCG
biotinylated sinR-D	bio-UCUCGGUAGAACCUCCA
Scrambled for sinR-T	bio-UCGUAACAGGUGGGCGGUAAAUG
biotinylated sinR-T	bio-GGGGAUAUAGCUCAGUGGUAGAGC

Supplementary Table 2) List of the oligonucleotides sequences used in the study.

Supplementary Table 3

Genes ID	Protein Descriptions	p-value	log2 Fold change	Fold change
Rbl2	Retinoblastoma-like protein 2	0,04	-0,74	-1,67
Oit3	Oncoprotein-induced transcript 3 protein	0,01	-0,53	-1,44
Sik2	Serine/threonine-protein kinase	0,00	-0,46	-1,37
Noct	Nocturnin	0,01	-0,46	-1,37
Nudt15	Nucleotide triphosphate diphosphatase NUDT15	0,01	-0,42	-1,34
C1rb;C1ra	Complement C1r-B subcomponent;Complement C1r-A subcomponent	0,03	-0,36	-1,29
Fgfr1	Fibroblast growth factor receptor 1	0,03	-0,33	-1,26
Mrpl19	Large ribosomal subunit protein bL19m	0,02	-0,3	-1,23
Sirt1	Sirtuin 1;NAD-dependent protein deacetylase sirtuin-1	0,04	-0,29	-1,22
Limk2	LIM domain kinase 2	0,01	-0,29	-1,22
Hmga1	High mobility group protein HMG-I/HMG-Y	0,05	-0,28	-1,22
Mylk	Myosin light chain kinase, smooth muscle	0,01	-0,28	-1,21
Rcc1l	RCC1-like G exchanging factor-like protein	0,02	-0,27	-1,21
Itgb2	Integrin beta;Integrin beta-2;Integrin beta	0,03	-0,27	-1,21
Sec61a2	SEC61 translocon subunit alpha 2	0,00	-0,27	-1,21
Polr1d	DNA-directed RNA polymerases I and III subunit RPAC2	0,03	-0,27	-1,20
Dnajc12	DnaJ homolog subfamily C member 12	0,02	0,26	1,20
Tnr	Tenascin-R	0,05	0,26	1,20
Helq	Helicase, POLQ-like (Fragment);Helicase POLQ-like	0,03	0,27	1,21
Hddc2	5'-deoxynucleotidase HDDC2	0,04	0,28	1,22
Fgd6	FYVE, RhoGEF and PH domain-containing protein 6	0,04	0,28	1,22
Nol8	Nucleolar protein 8	0,02	0,28	1,22
Copz1	Coatomer subunit zeta-1	0,00	0,29	1,22
Ttc19	Tetratricopeptide repeat protein 19, mitochondrial	0,04	0,29	1,22
Sumo1	Small ubiquitin-related modifier 1	0,00	0,29	1,22
Slc38a7	Sodium-coupled neutral amino acid transporter 7	0,01	0,30	1,23
Smad5	Mothers against decapentaplegic homolog 5	0,05	0,30	1,23
Dynlrb1	Dynein light chain roadblock-type 1	0,02	0,30	1,23
Cyb5b	Cytochrome b5 type B	0,05	0,32	1,25
Clpp	ATP-dependent Clp protease proteolytic subunit, mitochondrial	0,02	0,33	1,26
Atp8a2	Phospholipid-transporting ATPase IB	0,01	0,34	1,27
Tctn1	Tectonic-1	0,03	0,36	1,28
Papss2	3'-phosphoadenosine 5'-phosphosulfate synthase 2	0,03	0,36	1,29
Try10	trypsin	0,01	0,37	1,29
Paip2	Polyadenylate-binding protein-interacting protein 2	0,04	0,38	1,30
Cyb5a	Cytochrome b5	0,02	0,38	1,30
Ubtf	Upstream binding transcription factor, RNA polymerase I	0,05	0,41	1,33
Gstt3	Glutathione S-transferase theta-3	0,01	0,42	1,33
Ubqln2	Ubiquilin-2	0,02	0,42	1,34
Lage3	EKC/KEOPS complex subunit Lage3	0,05	0,42	1,34
Ufm1	Ubiquitin-fold modifier 1	0,05	0,42	1,34
Isoc2a	Isochorismatase domain-containing protein 2A	0,03	0,43	1,35
Wiz	Widely-interspaced zinc finger motifs	0,03	0,43	1,35
Ube2g1	Ubiquitin-conjugating enzyme E2 G1	0,00	0,46	1,38
Rfx5	DNA-binding protein Rfx5	0,03	0,47	1,38
Zkscan3	Zinc finger with KRAB and SCAN domains 3	0,05	0,50	1,41
Tm7sf3	Transmembrane 7 superfamily member 3	0,02	0,50	1,41
Slc1a4	Neutral amino acid transporter A	0,03	0,53	1,44
Dner	Delta and Notch-like epidermal growth factor-related receptor	0,04	0,56	1,47
Endov	Endonuclease V	0,03	0,88	1,83
Phf21a	PHD finger protein 21A	0,04	0,97	1,96
Ca4	Carbonic anhydrase 4	0,03	1,20	2,29
Lztr1	Leucine-zipper-like transcriptional regulator 1	0,03	1,35	2,55

Supplementary Table 3) List of differentially expressed proteins identified by mass spectrometry. Fold change >1.2, *p-value* <0.05

Supplementary Figure 1

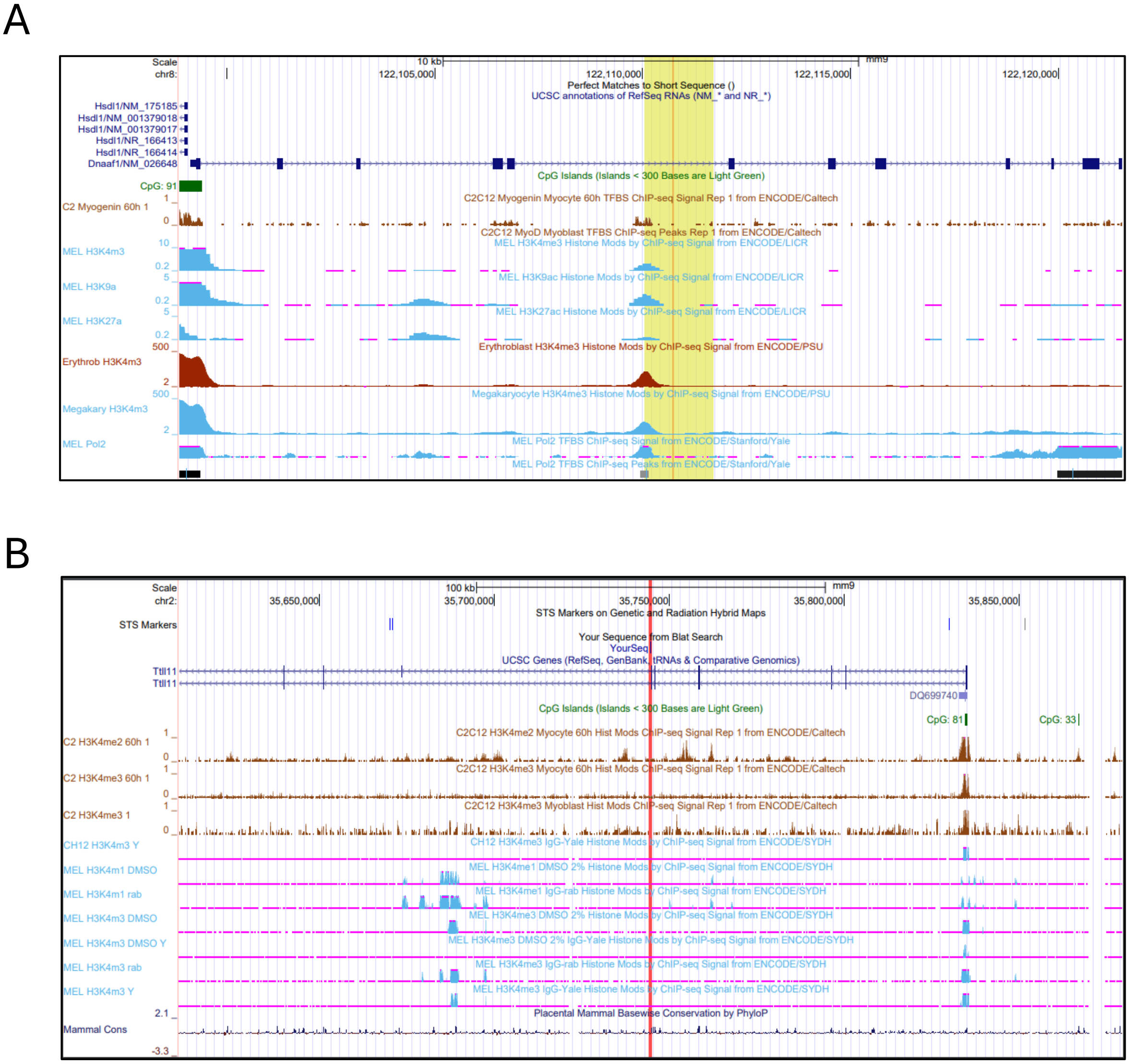

Supplementary Figure S1. Visualization of sinRNA locations using the UCSC Genome Browser. (a) sinR-D is mapped to intron 5 of the *dnaaf1* gene, indicated by an orange vertical line. The yellow segment highlights STARR-seq peaks, suggesting histone modifications at the start, indicative of a potential regulatory region. (b) sinR-T is located within an intron of the *TTLL11* gene, shown by a red vertical line, with histone modification peaks observed within the intronic region."

Supplementary Figure 2

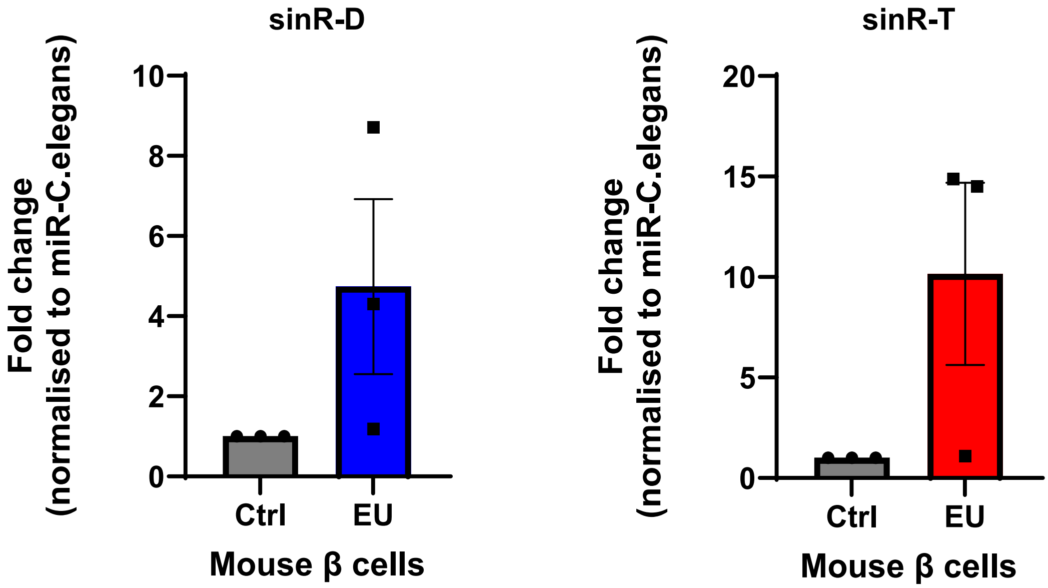

Supplementary Figure S2. qRT-PCR validation of small RNA-seq results. EU-tagged RNAs from CD4^{+} T lymphocytes were analyzed in beta cells three days post-adoptive transfer of immune cells. The qRT-PCR findings corroborate the RNA-seq data. The sequence of spike-in *C.elegans* miR-238 was used as normalisation control. Data are presented as mean ± SD, n=3.

Supplementary Figure 3

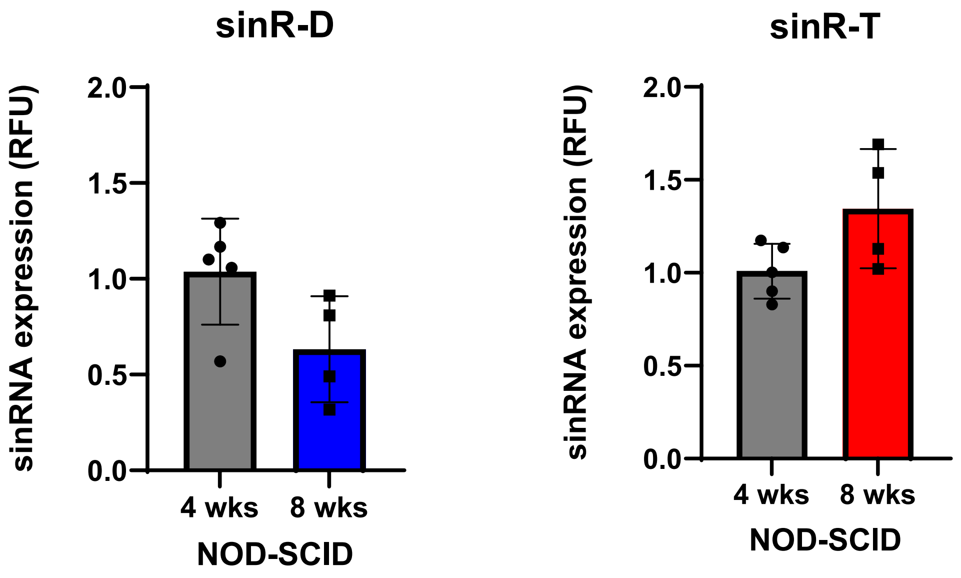

Supplementary Figure S3. **Levels of the selected sinRNAs in NOD-SCID mice islets.** sinRNA levels were quantified in NOD-SCID mice at 4 weeks and 8 weeks by qRT-PCR, Let7a was used for normalization, n=4-5, mean ± SD.

Supplementary Figure 4

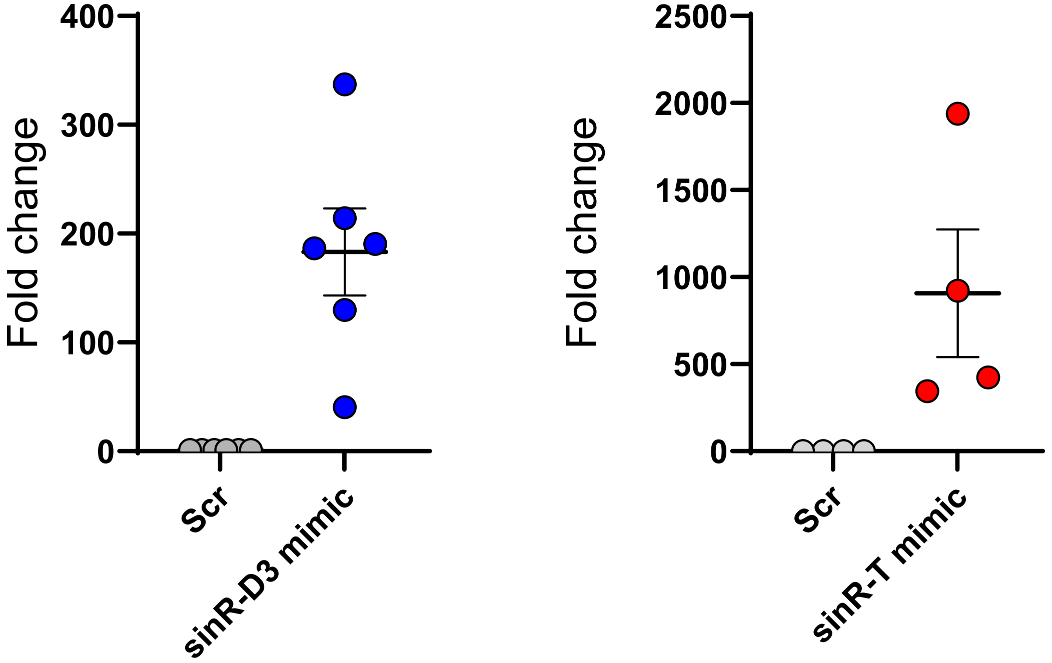

Supplementary Figure S4. **Overexpression of sinR-D3 and SinR-T using oligonlucleotide mimics. Dissociated pancreatic islet cells were transfected** with oligonucleotide mimicking the sequence of sinR-D3 (left panel) or sinR-T (right panel) or with their corresponding scrambled sequences (scr). The level of the sinRNAs was measured two days later by qRT-PCR. The level of the sinRNAs in cells transfected with the scrambled controls has been set to one.

Supplementary Figure 5

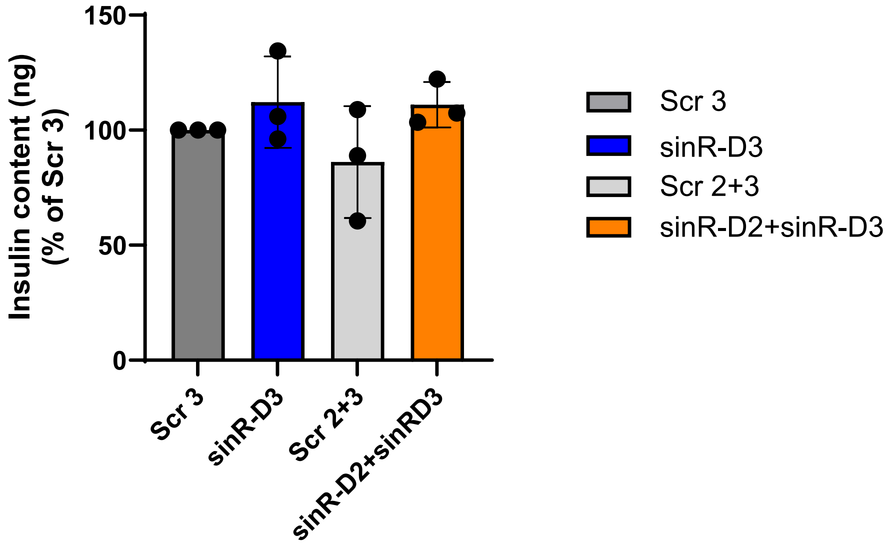

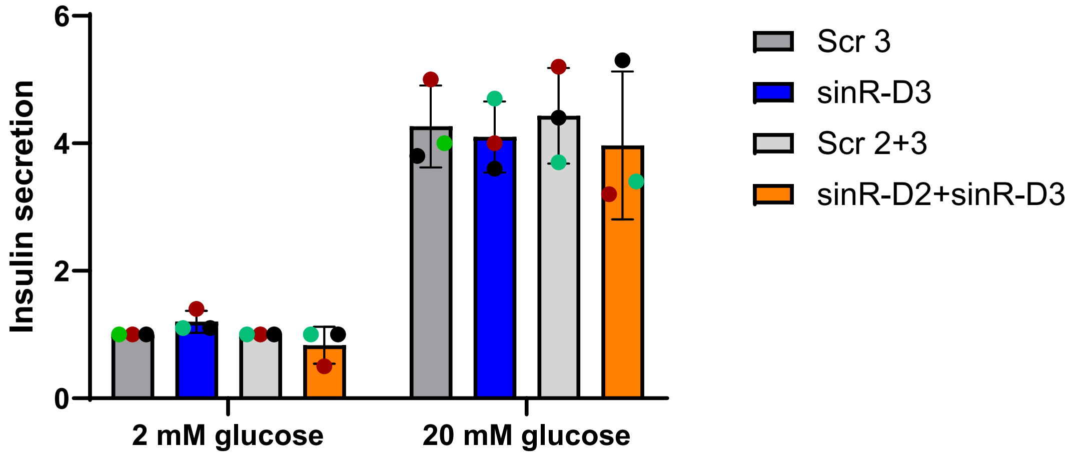

Supplementary Figure S5. **Impact of sinR-D2 and sinR-D3 on glucose-induced insulin secretion in the β-cell line MIN6.** The insulin-secreting mouse MIN cell line MIN6 was transfected with sinR-D3, with both sinR-D2 and sinR-D3 or with their corresponding scrambled sequences. Insulin content (upper panel) and insulin secretion in the presence of 2 or 20 mM were measured by ELISA).

Supplementary Figure 6

Schematic representation of pull-down assay

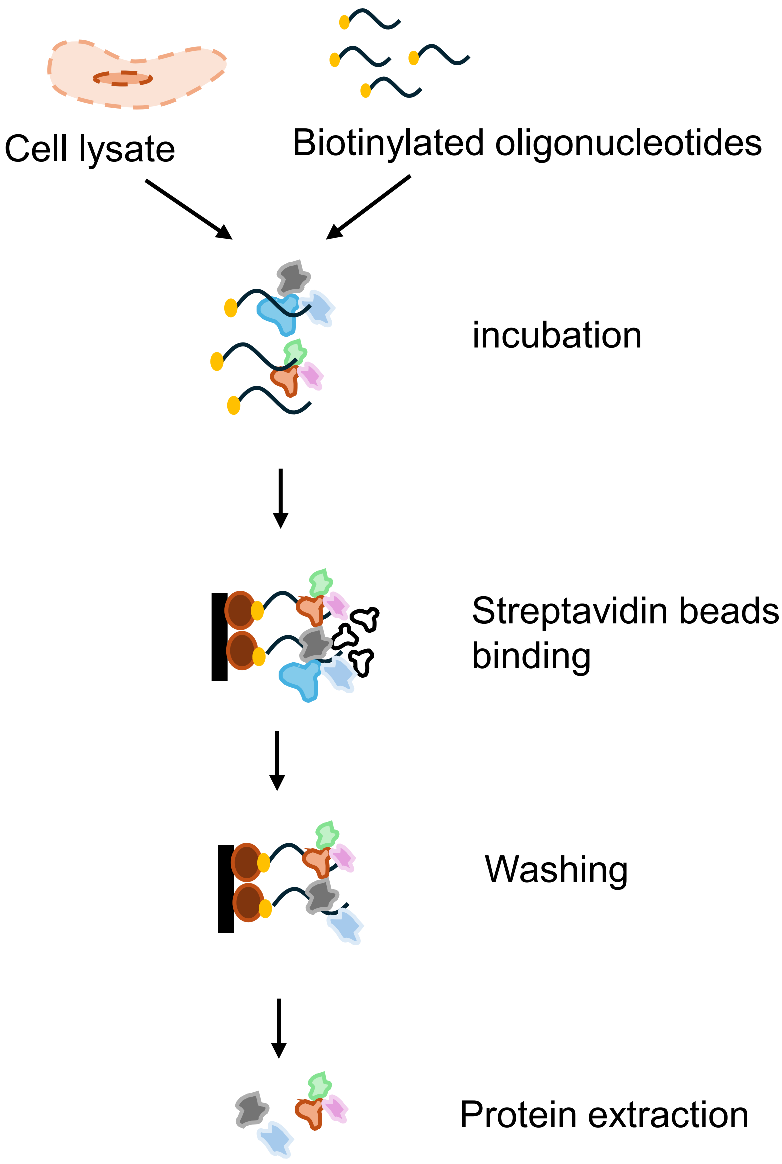

Supplementary Figure 7

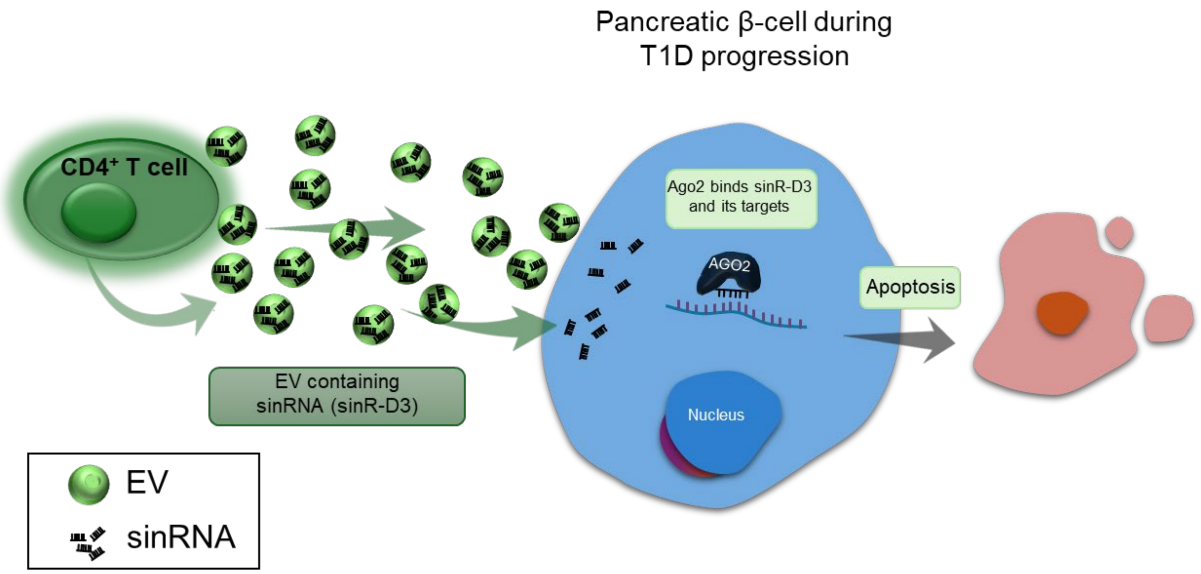

Supplementary Figure S7. Model describing the mode of action of sinR-D3 and its potential contribution to the initial phases of Type 1 diabetes

$CD4^+$ T cells infiltrating the islets release extracellular vesicles (EVs) containing the intron-derived small RNA sinR-D3. Upon uptake by pancreatic β-cells, sinR-D3 associates with AGO2 and represses target mRNAs, leading to the activation of apoptotic pathways and contributing to early β-cell loss during type 1 diabetes development.
